# Implementation of evidence-based clinical practice and its associated factors among health care workers at public hospitals in Sidama regional state, southern Ethiopia

**DOI:** 10.1371/journal.pone.0299452

**Published:** 2024-03-21

**Authors:** Tomas Yeheyis, Dawit Hoyiso, Yacob Abraham Borie, Negash Tagesse

**Affiliations:** 1 School of Nursing, Hawassa University, Hawassa, Sidama, Ethiopia; 2 School of Medicine, Hawassa University, Hawassa, Sidama, Ethiopia; PLOS, UNITED KINGDOM

## Abstract

**Background:**

Health disparities, inconsistent outcomes, and underwhelming health services continue to be problems for all nurses and doctors. Studies from a variety of nations have found that doctors and nurses have little familiarity with evidence-based practice. There is a knowledge vacuum about the degree of evidence-based nursing practice and its contributing factors in Ethiopia as well as the current study region.

**Objective:**

The study’s objective was to evaluate how healthcare professionals working in government hospitals in the Sidama regional state of 2022 are implementing evidence-based clinical practice and to identify associated factors.

**Methods:**

From October 1 to December 30/2022, a cross-sectional institution-based study with a mix of quantitative and qualitative methods was carried out. A total of 422 healthcare workers were randomly selected to participate in the current study from 6 randomly selected public hospitals in the region. This study included 25 key informant interviews. The Friedman test and Funk’s BARRIER scale were adapted into a semi-structured questionnaire quantitative. Multivariable logistic regression was used to assess the significance of the association between the dependent and independent variables. A pretest was conducted on 22 healthcare workers from Shashmane Hospital.

**Result:**

This study found that more than half, 51.7%, of the participants had a total implementation of evidence-based practice score below the mean score (40.4). only Access to the internet [having smart phone] (Adjusted Odds Ratio (AOR) = 3.03: 95% Confidence Interval (CI): (1.75–5.26))., favorable Attitude toward EBP of participants. (Adjusted Odds Ratio (AOR) = 1.84Confidence Interval (1.12–2.70)), inadequate Self-efficacy of Evidence-based practice skills (Adjusted Odds Ratio (AOR) = 0.29 Confidence interval = 0.19–0.46), and Knowledge of evidence-based practice (Adjusted Odds Ratio (AOR) = 0.45 Confidence interval = 0.30–0.77)were factors significantly associated with EBP implementation.

**Conclusion:**

More than half of the participants,51.7%, do not implement evidence-based practice in the care of their clients, which is unacceptable. The use of EBP has been significantly associated with access to the internet (having a smartphone), participants’ positive attitudes toward it, their level of skill efficacy in finding and accessing it, and their knowledge of it. Therefore, stakeholders ought to think about addressing these obstacles to the adoption of EBP.

## Introduction

Evidence-based practice (EBP) is the use of the best research finding (evidence) to answer a burning clinical question together with one’s clinical expertise generated from outcome management or quality improvement projects and patient preferences and values. Researchers generate new knowledge through rigorous research (external evidence) and evidence-based practice (EBP) provides clinicians the tools to translate the evidence into clinical practice and integrate it with internal evidence to improve the quality of health care and patient outcomes [1, 2]. Its basic principles are that practical decisions made should be based on research studies and that these research studies are selected and interpreted according to some specific norms and characteristics for evidence-based practice (EBP) [1, 3, 4].

Clinicians often ask how much and what type of evidence is needed to change practice. A good rule of thumb to answer this question is that there needs to be strong enough evidence to make a practice change. Specifically, the level of evidence plus the quality of evidence equals the strength of evidence, which provides clinicians the confidence that is needed to change clinical practice [1, 2].

The evidence-based practice utilizes the most up-to-date methods of providing care, which has been proven through the appraisal of high-quality studies and statistically significant research findings [2, 3].

Health care that is evidence-based and conducted in a caring context leads to better clinical decisions and patient outcomes. Gaining knowledge and skill in the evidence-based practice (EBP) process provides nurses, physicians, and other clinicians with the tools needed to take ownership of their practice. Evidence-based practice takes resources, work, time, and effort but the outcome makes them worthwhile. Every patient deserves care that is based on the best scientific knowledge and that ensures high-quality cost-effective care, higher job satisfaction, less staff turnover, and improved patient outcomes [3, 4].

For successful implementation of research that supports the effectiveness of a clinical intervention, evidence needs to be located of its highest quality. Successful implementation is a function of the relation between the nature of the evidence, the context in which the proposed change is to be implemented, and the mechanism by which the change is facilitated [4].

Despite all the programs and strategies to promote the use of research findings, there is still a gap between theory and practice. Many practices are being implemented in healthcare that have no or little evidence to support their use (e.g. double-checking of pediatric medication, routine assessment of vital signs every 2 or 4 hours in hospitalized patients [1, 4, 5]. This is indicative that there are limitations to EBP from becoming the standard of care throughout the world. Some studies from various countries have reported that nurses and physicians practice EBP and distinct EBP activities to a low extent [6–9].

All over the world, there is a growing appreciation of the importance and difficulty of evidence-based nursing practice (EBNP). As part of international efforts to facilitate the dissemination of EBNP, research has focused on identifying barriers to the utilization of EBNP. Identifying such barriers can help international efforts to develop strategies to overcome these [6, 10]. Though several key barriers to the utilization of research findings were discovered the work organization (setting), inadequate time for accessing and understanding research and for applying research to individual patients have been cited in several studies [5, 6, 10]. Many healthcare professionals have claimed that they lack the skills to evaluate the quality of evidence or the understanding of how to collect important information and limited access to information has also been identified as a concern in the implementation of EBP. Other challenges to EBP include inadequate data sources, apparent contradiction with patient choices, and a client’s fear of economic constraints [6, 11–13].

Even though evidence-based health care is an efficient and much-needed practice worldwide, developing countries have more difficulties in the accessibility of existing evidence, medical resources, and its implementation than developed countries.

In Africa, EBP implementation is late compared to the developed world. Incorporating evidence-based health care (EBHC) into the African context means setting priorities, developing evidence summaries and guidelines, and implementing research findings relevant to African countries to support health care for all. Contextualizing evidence relates to several issues, including the lack of evidence available for an African setting. The effectiveness of an intervention in Africa may be different from that found in studies elsewhere because of factors such as later presentation, co-infections, malnutrition, higher levels of self-medication, and use of traditional, reduced levels of resources, including human resources for basic health care, and political instability. In addition, effective interventions, as determined by many systematic reviews, may not be available or affordable in most African settings. This means that Africa needs valid African-specific research and that authors of systematic reviews should consider this by avoiding overgeneralization when making conclusions [4, 7].

Though EBP has been launched to be practiced in the country by the Federal Ministry of Health of Ethiopia recently, the ministry lacks skilled health professionals who could help to synthesize evidence for policy-making, a system for health professionals to access shreds of evidence and familiarity to the concept EBP by the healthcare providers. Moreover, at all levels of the health systems, there is little culture or tradition of trusting or using evidence [14].

There is a paucity of evidence and published research articles published concerning EBP utilization in Ethiopia. Therefore, this study intends to determine the level of EBP implementation and will give information concerning factors, which hinder it among nurses and physicians working at governmental hospitals in Sidama regional state.

## Materials and methods

### Study area

This study was conducted at governmental hospitals located in Sidama regional state. Sidama Regional State is the newly established state of the Southern Nation and Nationality People Regional State (SNNPR), its capital city Hawassa is 275 km. from Addis Ababa. There are 12 primary hospitals, four regional hospitals, and one referral hospital.

### Study design

Institution-based mixed study design (quantitative and qualitative) was employed from October 1 to December 30, 2022.

### Population

#### Source population

All nurses and physicians work at governmental hospitals in Sidama regional state.

#### Study population

All nurses and physicians working at selected governmental hospitals in Sidama regional state.

### Eligibility criteria

#### Inclusion criteria

All sampled nurses and physicians working at selected governmental hospitals in Sidama regional state who are available at the time of the data collection.

#### Exclusion criteria

Those who were seriously ill during the data collection period were excluded from the study.

### Sample size determination

The prevalence of evidence-based nursing practice at health facilities of Sidama regional state was calculated by using the single population proportion formula, assuming **P** = 50%, at the confidence level of 95% and marginal error of 5%, a total of 422 nurses and physicians will have participated.


$$n=\frac{{\left(Z_{\alpha /2}\right)}^{2}(pq)}{d2}$$

$$=\frac{{\left(1.96\right)}^{2}*(0.5*0.5)}{{\left(0.05\right)}^{2}}=384$$

= Non-respondent rate was taken at 10%

= Total sample size = **422**

where, q = 1-p, **n** = required sample size

### Dependent variables

Implementation of evidence-based practice

### Independent variables

A potential independent variable for the study has been identified after reviewing different literature [6, 8, 10, 11, 15].

### Socio-demographic factors

Age, sex, marital status, qualification, position in the organization, and level of hospital.

### Organizational factors

Administration support, colleague support, time availability, and incentive for staff.

### Characteristic of individual

Communication character, awareness of best evidence, knowledge of research evidence, salary, and place of graduation.

### Characteristics of research evidence

Way of presentation, understandability of research report, availability, and accessibility of research reports.

### Sampling technique and sampling procedure

Of the total number of 17 hospitals in the region 6 hospitals(Leku Primary Hospital, HUCS Hospital, Yirgalem General Hospital, Dore Primary Hospital, Yirba General Hospital, and Adare General Hospital) represent 30% of the population are selected randomly using a computer-generated random number. After the identification of the total number of nurses and physicians in the selected hospitals, the total sample size was allocated to each hospital using the proportional-to-size method.

### Data collection tool and procedure

#### Data collection instruments

*For quantitative data*. The data were collected using a pre-tested semi-structured questionnaire adapted version of Funk’s BARRIERS scale to measure nurses’ perceived barrier to research utilization and Friedman’s test will be used to measure the knowledge of respondents [16].

This questionnaire will include the 29-item barrier scale to research utilization. This scale asks the nurses and physicians to rate the extent to which they supposed each item as a barrier to their use of research evidence to change or improve their practice.

The questionnaire was prepared in the English language. The first part of the questionnaire contained items, which measure the socio-demographic characteristic of the respondents. The second part of the questionnaire contained questions about awareness and knowledge of evidence-based nursing practice. The third part of the questionnaire contained questions about evidence-based practice activities. The fourth part of the questionnaire contained questions about the source of information and availability of information resources. Part five of the questionnaire contained the ’BARRIER’ scale, which measured the nurse’s perception of barriers to research evidence utilization. The ’BARRIER’ scale is divided into four parts (subscales). Characteristics of the adopter(nurse’s value, skills, and awareness), characteristics of the organization (setting, barriers, and limitations), characteristics of the innovation (qualities of the research), and characteristics of the communication (presentation and accessibility). The tool is valid and reliable with Cronbach’s alphas were ≥.65. Pearson correlation estimates of test-retest reliability ranged from .68 to .83

*For qualitative data*. A Key Informant interview was conducted with an adequate number of individuals physicians and nurses who have tremendous clinical experience, coordinate case teams, and are believed to have rich information. Five Msc. Holders experienced in data collection interviewed all the interviewees and took hand notes and tape records.

### Data collectors

Five BSc nurses were recruited to facilitate data collection. Data collectors were trained for two days intensively on the study, the confidentiality of the information, and informed consent.

### Data quality control

The questionnaire was adapted from valid sources (Funk’s BARRIERs scale and Friedman’s test). Trained data collectors were facilitating data collection after a pre-test had been done on 11 nurses and 11 physicians of Shashmane Hospital. Correction on the questionnaire was done after the pre-test the questionnaire. Data collectors were supervised in issues of correct data collection procedures. To ensure anonymity, code numbers were placed on the completed questionnaire after they were returned to the investigators.

### Data quality assurance for qualitative data

The trustworthiness of qualitative data was enhanced by preventing the premature closing of interviews.

### Data processing and analysis

The quantitative data were carefully entered into Epi-data V 4.6.0.6 edited, and cleaned for inconsistencies and missing values. Data were analyzed using SPSS version 25. Descriptive statistic was used to summarize data. Simple and multivariable logistic regression was used to assess the association between the dependent variable and independent variables. A P-value less than 0.05 and CI that doesn’t cross the null value (AOR of 1) were taken to declare the significance of the association and a point estimate of AOR and respective confidence intervals are used in discussing the findings of the study. The necessary assumption of logistic regression, and model fitness was checked using Hosmer and Lemeshow’s goodness of fittest statistics. Multi-collinearity was checked by a variance inflation factor (VIF) that shows the collinearity of each variable in this study was less than 2.5.

The qualitative data analysis was carried out simultaneously with data collection. The audio-recorded interview was transcribed verbatim immediately on the same day, and it was translated into the English language. The translated data was read line-by-line to understand the context and the meanings. In the process, data were exported to ATLAS. ti 7.1 software to produce meaningful codes and do further analysis. The initial codes were subjected to discussion and refinement throughout the data coding period as new codes emerged from the text. Multiple coders have participated in the coding process and categorization of the codes. These codes were merged into larger categories and then set into sub-themes. Sub-themes were reviewed further to develop overarching themes. Then the data were summarized and reported with some important direct quotes.

### Operational definition

**Good Knowledge:** Those respondents who scored above the mean for knowledge questions.

**Poor Knowledge**: Those respondents who scored below the mean for knowledge questions.

**Favorable Attitude**: respondents who scored above the mean score for attitude questions.

**Unfavorable Attitude**: Respondents who scored below the mean score for attitude questions.

**Good level of implementation of EBP**: Respondents who scored above the mean score for implementation questions.

**Poor Level of implementation of EBP**: Respondents who scored below the mean score for implementation questions.

### Ethical clearance

Ethical approval and clearance were obtained from the institutional review board of Hawassa University and permission to conduct the study was obtained from the health bureau of the city and management of the respective health institutions. Written informed consent was obtained from the respondents before participation. After all permission and the necessary budget were secured, a letter of permission was submitted to the concerned bodies. Confidentiality was assured throughout the study by the use of code numbers instead of individual names for identification.

All participants were reassured that they could withdraw from the research at any time if they felt uncomfortable with the study.

All findings and results were presented as facts stated in the interviews. All participants’ experiences and perceptions were portrayed as they did so in the interviews, and no false information or accusations were included in the final report.

## Result

### Socio-demographic characteristic

In this study, 422 respondents participated giving a response rate of 100%. Out of these participants, the majority 271(64.2%) were female, married 260(61.6%), 214(50.7%) were protestant followed by 175(41.5%) orthodox. Also majority, 386(91.5%) were urban residents. “Table 1”.

**Table 1:** Socio-demographic characteristics of health care workers at public hospitals in Sidama regional state, southern Ethiopia, 2022.

Socio-demographic variables		Frequency	Percent
**sex**	Male	151	35.7
	Female	271	64.3
**Marital status**	Single	157	37.2
	Married[ever-married]	265	62.8
**Religion**	Protestant	214	50.7
	Orthodox	175	41.5
	Muslim	27	6
	Others	3	0.7
**current Working unit**	Adult OPD	52	12.3
	Pediatric OPD	33	8
	Medical ward	31	7
	Surgical ward	54	12.7
	Gynecology and obstetrics ward	93	22
	Pediatric ward	43	10
	ART clinic	6	1.4
	Emergency OPD	110	26.1
**profession**	Medical doctors	84	20
	Nursing	338	80
**Working hours**	≤8	413	97.8
	>8≤16	9	2.2
**Working area**	Urban	386	91.5
	Rural	36	8.5

### Knowledge about evidence-based healthcare practice

25 items were used to assess the level of knowledge of the participants of the study about evidence-based healthcare practice. Among all participants in the study, 309(73.2%) had heard about the term ’evidence-based practice or related terms’ in their practical life. Regarding the hierarchy of evidence, 183 (43.4%) respondents indicated that the evidence from systematic review reports is the uppermost in the hierarchy, while 86 (20.4%) participants indicated that reports from meta-analyses are uppermost in their quality hierarchy. Concerning their consideration of the levels of evidence they read 296(70.1%) reported that they consider the level of the evidence before reading. Regarding the use of evidence-based clinical practice98 (23.2%) reported is as it is important to improve the quality of care, and 38(9.0%) reported increasing patient satisfaction. 213(50.5%) reported that they Can identify a site where clinical journals, articles, and guidelines are published. The majority, 295(69.9%) perceive that critically appraising evidence is an important step in evidence-based practice, 201(47.6%) reported that they had difficulty understanding research reports, 246(58.3%) knew how to implement EBP sufficiently to change practice, When we considered the overall summary, 241 (57.1%) of the participants scored below the mean score and were poorly knowledgeable. “Table 2”.

**Table 2:** Knowledge of evidence-based health care practice among health care workers at public hospitals in Sidama regional state, southern Ethiopia, 2022.

	Knowledge questions	Response category	N	%
1	Have you heard of the term evidence-based practice or related terms?	Yes	309	73.2
		No	113	26.8
2	types of studies uppermost in the hierarchy of evidence	Systematic review	183	43.4
		Meta-analysis	86	20.4
		Both	153	36.3
3	Do you take into consideration the levels of evidence at usage	Yes	296	70.1
		No	125	29.6
4	The use of evidence-based clinical practice is	Improve quality	98	23.2
		Increase patient satisfaction	38	9.0
		All	281	66.6
5	Can you identify a site where clinical journals, articles, and guidelines are published	Yes	213	50.5
		No	205	48.6
6	Critically appraising evidence is an important	Yes	295	69.9
		No	125	29.6
7	Do you have difficulty understanding research reports?	Yes	201	47.6
		No	219	51.9
8	Do you know how to implement EBP sufficiently change?	Yes	246	58.3
		No	174	41.2
9	Do you use a smartphone?	Yes	322	76.3
		No	98	23.2
10	Do you use smartphones for searching best evidence to improve clinical practice?	Yes	300	71.1
		No	120	28.4
		No	150	35.5

According to key informant interview findings, Some had mentioned Evidence-based practice as "Care based on new evidence from guidelines and other sources of evidence" OneOne KI, an Emergency case team coordinator nurse from a general hospital, has mentioned that he is not familiar with the term Evidence-based care as

“….*ok I am not familiar but*… *I think evidence-based clinical practice means providing patients with as much information as possible about their clinical condition*”.

### Attitude towards EBP

Our analysis of respondents’ perceptions of evidence-based practice (EBP) in the research domain yielded the following conclusions. We combined "Strongly disagree" and "disagree" to become "disagree," and we combined "agree" and "strongly agree" to form "agree." The total score, which ranged from 9 to 35, was calculated. We also assessed the respondent’s attitude toward evidence-based practice’s mean and median scores. Therefore, the median score was 25, with an IQR of 20–27, while the overall mean score was 23. According to this score, respondents defined as having an unfavorable attitude were those who scored below the mean while respondents classed as having a positive attitude were those who scored above the mean. Accordingly, 232(55.9%) had a favorable attitude toward evidence-based healthcare practice. "Table 3".

**Table 3:** Attitude towards evidence-based health care practice among health care workers at public hospitals in Sidama regional state, southern Ethiopia, 2022.

	Attitude Questions	Disagree N (%)	Agree N (%)
1	Implementing evidence-based healthcare practice will improve the care that I deliver to my patients	78**(**18.5)	256(60.7)
2	Evidence-based practice is not relevant to my profession	58(13.74)	284(67.3)
3	Training should be given about evidence-based healthcare practice	139(32.9)	224(53.0)
4	Recent national guidelines improve clinical care	80(19.0)	246(58.3)
5	EBP takes too much time so it is difficult to implement	208(49.3)	90(21.3)
6	Research findings are useful in my day-to-day care of patients	106(25.1)	227(53.7)
7	I feel that Most of my clinical practice is currently evidence-based	120(28.4)	179(42.5)

However, in qualitative data, some of the interviewees in in-depth interviews mentioned that Evidence-based practice increases the quality of care provided to the clients.

One of these KIs, a physician in a general hospital, stated

“…*I do think Evidence-based practice is very important because*, *in my short career*, *I have seen a lot of changes regarding management guidelines of different conditions*. *Therefore it is very important to apply evidence for care for both the clients and the practitioner himself*. *In addition*, *it encourages one to develop oneself professionally*. *From my perspective evidence-based practice will increase patient care and satisfaction*, *on the other hand*, *it helps in proper utilization of resources as contemporary procedures use minimum resources*.”

### Skills in evidence-based practice

Regarding their abilities to use evidence-based practice, 274 (64.9%) said they could find the best research to address pressing clinical questions, 288 (68.1%) said they could formulate a clinical question based on a particular patient problem, 223 (52.8%) said they could find the best resources for doing so, and 265 (62.8%) said they could put research-based recommendations into practice. However, after calculating or adding up the entire self-efficacy score of nurses and doctors for implementing evidence-based practice, it was discovered that 176 (41.7%) of them were unable to carry out actions that were beneficial for EBP implementation. Participants who responded “Yes” to each of the six questions assessing the skill are awarded one point for each question while they will not get a point for the question they responded “NO”. The median and IQR results were 4 and 3–5 respectively. “Table 4”.

**Table 4:** Skills in using evidence-based health care practice among health care workers at public hospitals in Sidama regional state, southern Ethiopia, 2022.

N^o^	Skill Questions	Responses		
			No	%
1.	Can you search for the best evidence to answer clinical questions in a time-efficient way?	Yes No	274 148	64.9 35.1
2.	Can you formulate a clear question based on a specific patient problem?	Yes No	288 134	68.1 31.8
3.	Can you access the best resources to implement evidence-based practice?	Yes No	223 199	52.8 47.2
4.	Do you have the ability to implement recommendations of research studies into clinical practice?	Yes No	265 157	62.8 37.2
5.	Do you have the ability to find appropriate research reports?	Yes No	255 167	60.4 39.6
6.	Do you feel confident in judging the quality of research reports?	Yes No	231 191	54.7 45.3

### Level of implementation of evidence-based practice

Each of the 18 items used to assess the level of implementation of evidence-based practice was scored from 1 to 5. In this regard, the participants have a score ranging from 18 to 90. The mean, median, and IQR of IEBP scores were 40.4, 40, and 28–50 respectively. Nearly half, 51.7%, of the respondents have scored above the mean value showing a good level of implementation of EBP.

There was the highest mean score for the two factors," How often do you Evaluate patient outcomes after practice change during the last two months?" and "How often have you Accessed Up-to-date during the last 2 months?" “Table 5”.

**Table 5:** Implementation of evidence-based health care practice among health care workers at public hospitals in Sidama regional state, southern Ethiopia, 2022.

	EBP implementation during the past 2 months	Mean	SD
1	How often you had Collected data and formulated a clinical question during the last two months?	1.94	1.039
2	How often you had applied findings of clinical literature into clinical practice during the last two months?	2.24	1.143
3	How often you had Looked at recent national guidelines and new treatment protocols during the last two months?	2.62	1.318
4	How often you had Used a recent guideline to change practice during the last two months?	2.31	1.209
5	How often you had Added new types of healthcare based on literature during the last two months?	2.21	1.211
6	How often you had Changed care based on a discussion with a patient/family member during the last two months?	2.26	1.200
7	How often you had Changed practice based on information received from in-service during the last two months?	2.10	1.084
8	How often you had Accessed Up-to-date during the last two months?	2.36	1.252
9	How often you had Accessed PubMed during the last two months?	2.16	1.214
10	How often you had Accessed HINARI during the last two months?	2.01	1.160

In A qualitative analysis, five out of eight KI mentioned they implement evidence into care practice as much as possible but three KI has mentioned because of several reasons evidence-based care practice is not well implemented.

One of these three KIs stated, "*Frankly regarding practice or application of this evidence is possible and we are trying it but I cannot say that we are implementing the best evidence we are getting from different sources*.”

### Barriers to evidence-based practice

Respondents of the study were asked 17 questions to identify barriers to the implementation of EBP in their setting. Among the major barriers identified in the study; two-thirds of participants in the study reported a Lack of training about EBP which makes it difficult to implement. Accordingly, 59.4% of the respondents in the study indicated that it is difficult to find recent national treatment guidelines and protocols. On the other hand, 64.2% of the respondents agreed that Patient illiteracy makes it difficult to discuss management information. Similarly, 69.3% and 69.6% of the study participants agreed that Low patient awareness about their disease management makes it difficult to implement EBP, and the Lack of an organized patient education department makes it difficult to incorporate patient preferences into practice. Accordingly, 62.2% of the respondents agreed that there is a lack of regular orientation about new health priority issues Among all participants to whom the question "An absence of interdisciplinary discussion during patient management” is posed 72.1% agreed that the absence of interdisciplinary discussion in patient management is a barrier in the implementation of EBP. “Table 6”.

**Table 6:** Barriers to evidence-based practice reported by health care workers at public hospitals in Sidama regional state, southern Ethiopia, 2022.

	Barrier Items	A proportion of participants agreed	
		No	%
1	Hospital infrastructure, such as computers, Internet, and new treatment guidelines, are not adequate for the implementation of EBP	170	41
2	There is a Lack of training about EBP which makes it difficult to implement	272	66.2
3	It is difficult to find recent national treatment guidelines and protocols	244	59.4
4	Problems understanding English make it difficult to use the literature	198	48.2
5	Patient illiteracy makes it difficult to discuss information regarding management	264	64.2
6	Low patient awareness about their disease management makes it difficult to implement EBP	285	69.3
7	The lack of an organized patient education department makes it difficult to incorporate patient preferences into the practice	286	69.6
8	There is no sufficient information to find new guidelines/protocols	199	48.4
9	There is a lack of regular orientation about new health priority issues	255	62.2
10	There is no sufficient time to find new guidelines/protocols or online research findings	197	48
11	It is difficult to understand national treatment guidelines and protocols	205	50
12	The culture of our team is not receptive to changing practice	230	56.1
13	There is a lack of authority in the workplace to change the practice	236	57.6
14	Hospital managers are not supportive of EBP	206	50.2
15	An absence of interdisciplinary discussion during patient management	302	72.1
16	The importance of evidence for practice is not made clear	226	55.1
17	Team managers do not initiate the use of evidence-based practice	206	50.2

The qualitative study has revealed the above-mentioned and other additional barriers to the implementation of evidence-based practice.

One of the key informants, an emergency team coordinator medical doctor states

“…well *we face several limitations in the implementation of EBP*. *One is since our hospital is not an academic area you may not get a chance to transfer all the updated information you get to your colleagues*. *Other than this shortage of necessary equipment for the implementation of the evidence*, *even the absence of enough space for providing health education and even the absence support of from the management are general limitations*. *To be more specific for example there are potent drugs for specific diseases that we need to prescribe to the clients which you know will result in a better result but these drugs are inaccessible as well unaffordable by the patients*. *Similarly*, *you see new practices but we don’t have accessible resources*. *For example*, *As a developing country our biggest burden is communicable diseases*, *and to screen these we need a culture which is not available here*.”

Similarly, the other Key informant, the Chronic cases follow-up team leader, stated

“…*ok as an obstacle our source of evidence is mainly limited to the evidence which comes through the hospital nursing service*. *So sometimes the nursing service may not provide as much updated evidence as possible*. *The other obstacle is the lack of internet connection in the hospital*, *similarly lack of drugs and other resources needed for the management of cases*. *Otherwise*, *lack of information and training on the evidence-based practice itself is a challenge*.”

### Factors associated with the implementation of evidence-based practice

After adjusting for potential confounders in multivariable logistic regression analysis; only Access to the internet [having a smart smartphone], favorable Attitude towards EBP of participants, adequate Self-efficacy of EBP skills, current profession, and Good Knowledge of EBP were significantly associated with EBP implementation. Age and other sociodemographic and institution-related characteristics weren’t significantly associated with EBP implementation in multivariate analysis.

The odds of EBP implementation increase by 3.41 times among participants with adequate Self-efficacy of IEBP skills compared to their counterparts (AOR = 3.41(2.08–5.01)). The Odds of EBP implementation were 1.58 times higher among participants having Access to the internet [having a smart smartphone] (AOR = 1.58(1.06–2.18)).

This finding is congruent with the finding from the qualitative study where one of the KIs stated “…….*yaa other than the other opportunities I mentioned earlier availability of internet in the compound is an opportunity in accessing and implementing best evidence*." On the other hand, the other KI states "*ok as an obstacle our source of evidence is mainly limited to the evidence which comes through the hospital nursing service*, *more over the other obstacle is the lack of internet connection in the hospital*, *which adds the reason for lack of evidence for providing evidence-based care to the clients*.”

The odds of EBP implementation were 2.01 times higher among participants having favorable Attitudes toward EBP implementation compared to Participants with unfavorable attitudes towards EBP implementation. (AOR = 2.01[1.13–3.21)) The Odds of EBP implementation increased by 1.68-fold among participants having good knowledge about EBP than those having poor knowledge (AOR = 1.68(1.12–1.87)). “Table 7”.

**Table 7:** Factors associated with evidence-based practice implementation among health care workers at public hospitals in Sidama regional state, southern Ethiopia.

Variables	Category	IEBP		AOR (95% CI)
		Good	Poor	
Access to the Internet [having smart phone]	Yes	143	129	1.58(1.06–2.18)*
	No	61	87	
Attitude towards EBP	Favorable	137	95	2.01[1.13–3.21]*
	unfavorable	93	89	1
Self-efficacy of IEBP skills	Adequate	150	96	3.41(2.08–5.01)*
	inadequate	54	122	1
Knowledge on EBP	Poor	99	76	1
	Good	105	136	1.68(1.12–1.87)

*P = <0.05, IEBP = implementation of EBP

## Discussion

This study identified that 51.7% of the participants did not implement EBP adequately which means, that 218(51.7%) of the participants scored below the mean score (40.4). this finding was lower compared to a study done in northern Ethiopia by Dagne et al where About two-thirds of the participants had a total IEBP score below 60% and a study done among nurses in Tikur and hospital Addis Ababa at 57.6% [8, 11]. However, this finding was higher compared to a national cross-sectional study among nurses in Sweden at 30%, a study done in the UK among midwives regarding implementation of evidence in the management of birth-related perineal trauma at 6% [6, 8]. These differences in the level of implementation of EBP can be attributed to the difference in methods of assessment and the population of the study.

The EBP implementation was positively associated with Access to the internet [having smart phone], favorable Attitude towards EBP of participants, adequate Self-efficacy of EBP skills, current profession, and Good Knowledge of EBP.

The finding from the multivariable analysis showed that The odds of EBP implementation increased by 3.41 times among participants with adequate Self-efficacy of IEBP skills compared to their counterparts (AOR = 3.41(2.08–5.01)). This is congruent with a study conducted among nurses and midwives in Amhara region hospitals in Ethiopia and a nationwide study conducted among nurses in Sweden where self-efficacy(capability belief) increases the odds of implementing EBP [6, 11]. Similarly, a study conducted on Norwegian nurses shows that Self-reported skills in finding, reviewing, and using different sources of evidence were positively associated with the use of research evidence [17]. This can be attributed to the fact that those health workers having adequate skills in searching and accessing evidence will have a higher chance of implementing the best available evidence into clinical practice. Incorporating evidence-searching courses in clinical education and continuous staff development have a lied foundation for EBP in different countries by raising nurses’ and physicians’ ability to search, appraise, and usage of the best evidence available [18]. The Odds of EBP implementation were 1.58 times higher among participants having Access to the internet [having a smart smartphone] (AOR = 1.58(1.06–2.18)). A study conducted among health professionals working in their hospitals in northern Gondar, Ethiopia found that Internet access is positively associated with the implementation of EBP [AOR: 1.831, 95% CI = (1.191–2.816)] [8]. Similarly, a study conducted in China found that Nurses who have access to more available resources, such as electronic databases, libraries, and professional guidelines, tend to rely more on scientific evidence [19]. Even though the evidence is released in the form of published hard copies these days access to the internet is the main gateway to accessing the best evidence for clinical practice. Those PR actioners who access the internet through their smartphones or computers will have easy access to the internet. This intern will give them a chance to browse the internet for the best evidence and implement them. Besides, those having a smartphone and access to the internet will have an increased chance of using applications that give access to updated evidence [20].

The odds of EBP implementation were 2.01 times higher among participants having favorable Attitudes toward EBP implementation compared to Participants with unfavorable attitudes towards EBP implementation. (AOR = 2.01[1.13–3.21)). This finding is in line with a study conducted among nurses and midwives in Amhara region hospitals in Ethiopia where the favorable attitude of participants increases the odds of implementation of EBP (AOR = 5.02, CI 1.2–21.5) [11]. Similarly, a study conducted in Oman shows that those nurses and midwives who have less positive attitudes towards EBP and limited EBP knowledge are less likely to implement EBP [21]. Attitude is the way of thinking or feeling about something, which determines your action toward the implementation or abstains from doing so. A favorable attitude towards an issue is a precursor for taking action in favor of the issue. In this regard, those participants of the study who have a favorable attitude will be keen to implement the evidence they have accessed from different sources [22].

The Odds of EBP implementation increased by 1.68-fold among participants having good knowledge about EBP than those having poor knowledge (AOR = 1.68(1.12–1.87)). This finding is in line with a study conducted among nurses and midwives in Amhara region hospitals in Ethiopia where knowledge of participants increases the odds of implementation of EBP (AOR = 3.06, CI 1.6–5.77) [11]. Similarly, a study in Tikur Anbesa Hospital, Addis Ababa finds out that those who are Knowledgeable have increased odds of implementing EBP(AOR 3.2 95%CI 1.5–7.0) [8] Awareness developed from educational sessions that Incorporated EBP (either in curriculums or through continuing education), knowledge of technical terms from the personal effort of reading and improved skills are needed to retrieve and critically appraise information and evidence. Having good knowledge of EBP also having increased awareness of EBP and its advantages of EBP in the care practice of their clients. This in turn plays a positive role in the clinicians’ attitude and ability to the implementation of EBP to increase the quality of care practice for clients [13].

Among the major barriers, this study found in the implementation of EBP is that two-thirds of participants in the study reported a Lack of training about EBP which makes it difficult to implement. Similarly, in a study done in three hospitals in Gondar, Ethiopia health professionals have indicated a lack of training as a barrier to the implementation of EBP [23]. Accordingly, 59.4% of the respondents in the study indicated that it is difficult to find recent national treatment guidelines and protocols. A similar study in China states that a lack of both reading and implementation resources were barriers to the implementation of EBP [19]. This finding is also supported by a study conducted among nurses in Norway where a significant number of nurses do not know how to find organizational information (protocols, guidelines) [17].

Similarly, 69.3% and 69.6% of the study participants agreed that low patient awareness about their disease management makes it difficult to implement EBP and the Lack of an organized patient education department makes it difficult to incorporate patient preferences into practice.

Accordingly, 62.2% of the respondents agreed that there is a lack of regular orientation about new health priority issues. This is in line with a nationwide survey conducted among medical, nursing, pharmacological, and allied healthcare professionals in Taiwan [15].

On the other hand among all participants of the study to whom the question "An absence of interdisciplinary discussion during patient management" is posed 72.1% agreed that the absence of interdisciplinary discussion in patient management is a barrier to the implementation of EBP. Similarly, the study from Norway found that nurses who participated in the study mentioned a less collaborative culture of their team as a barrier to changing the current practice into EBP [17]. This finding is also supported by another study conducted among nurses which finds out that the current culture and interaction among nurses themselves and other professionals do not support the implementation of EBP in the care of their clients [24].

## Conclusion

More than half of the participants 51.7% do not implement evidence-based practice in the care of their clients. Therefore, evidence-based clinical practice needs to be enhanced to improve clinical treatment. The use of EBP has been significantly associated with access to the internet (having a smartphone), participants’ positive attitudes toward it, their level of skill efficacy in finding and accessing it, and their knowledge of it. Therefore, stakeholders ought to work on addressing these obstacles to the adoption of EBP.

### Strengths and limitations of the study

#### Strength

- ✓ A standard data collection tool was used to collect data.
- ✓ To reduce bias, day-to-day supervision was carried out during data collection.

#### Limitations of the study

✓ Because it was a cross-sectional study it may not show cause and effect relationship

## Supporting information

S1 Dataset Reproducible dataset on the level of implementation evidence-based practice among health care workers at public hospitals in Sidama regional state, southern Ethiopia. (SAV)

## References

[1] Melnyk BM. Evidence-based practice in nursing & health care, a guide to best practice. 2004

[2] Mayer D. Evidence‐based Medicine. Epilepsia. 2006 Oct;47:3–5. doi: 10.1111/j.1528-1167.2006.00651.x 17044817

[3] Beyea SC, Slattery MJ. Evidence-based practice in nursing. A guide to successful implementation; 2006: p11

[4] Kitson A, Harvey G, McCormack B. Enabling the implementation of evidence-based practice/: a conceptual framework. 1998; 149–58. 10.1136/qshc.7.3.149 PMC2483604 10185141

[5] Penagiari D. et al, barriers and facilitators for implementation of evidence-based practice among Germany nurses working in a general hospital. 2008.

[6] Bostrom A, Rudman A, Ehrenberg A, Gustavsson JP, Wallin L. Factors associated with evidence-based practice among registered nurses in Sweden/: a national cross-sectional study, 2013 10.1186/1472-6963-13-165 PMC3648399 23642173

[7] Wikipedia [Internet] 2014 available from: http//en.wikipedia.org/wiki/evidence-based_practice Importance of EBP in Africa [Internet], 2015 available from: http://ebm.bmj.com/content/early/2013/02/20/eb-2012-101143

[8] Hadgu G, Almaz S, Tsehay SJ, Almaz S, Tsehay S. Assessment of nurses’ perceptions and barriers on evidence-based practice in Tikur Anbessa specialized hospital Addis Ababa Ethiopia. Am J Nurs Sci. 2015;4(3):73–83

[9] Melnyk BM, Fineout‐Overholt E, Giggleman M, Choy K. A test of the ARCC© model improves implementation of evidence‐based practice, healthcare culture, and patient outcomes. Worldviews on Evidence‐Based Nursing. 2017 Feb;14(1):5–9. 28002651 10.1111/wvn.12188

[10] Eizenberg MM. Implementation of evidence-based nursing practice: nurses personal and professional factors; J Adv Nurs[Internet], 2011. Jan[cited 2014Dec 5];(1):33–42 doi: 10.1111/j.1365-2648.2010.05488.x 20969620

[11] Dagne AH, Beshah MH, Kassa BG, Dagnaw EH. Implementation of evidence-based practice and associated factors among nurses and midwives working in Amhara Region government hospitals: a cross-sectional study. Reproductive Health. 2021 Dec;18(1):1–0. 33579309 10.1186/s12978-021-01096-w PMC7881559

[12] Bick DE, Ismail KM, Macdonald S, Thomas P, Tohill S, Kettle C. How good are we at implementing evidence to support the management of birth-related perineal trauma? A UK-wide survey of midwifery practice. BMC pregnancy and childbirth. 2012 Dec;12(1):1–0. doi: 10.1186/1471-2393-12-57 22731799 PMC3472238

[13] Jette DU, Bacon K, Batty C, Carlson M, Ferland A, Hemingway RD, et al. Evidence-based practice: beliefs, attitudes, knowledge, and behaviors of physical therapists. Physical therapy. 2003 Sep 1;83(9):786–805. 12940766

[14] Gautham M, Birhanu D, Umar N, Ghosh A, Elias N, Spicer N, et al. Panel discussion. The challenges of translating evidence into policy and practice for maternal and newborn health in Ethiopia, Nigeria, and India. BMC Health Serv, res.[Internet].

[15] Weng Y.H., Kuo K.N., Yang C.Y., Lo H.L., Chen C. and Chiu Y.W., 2013. Implementation of evidence-based practice across medical, nursing, pharmacological and allied healthcare professionals: a questionnaire survey in nationwide hospital settings. Implementation Science, 8(1), pp.1–10. doi: 10.1186/1748-5908-8-112 24063756 PMC3849261

[16] Funk SG, Champagne M, Wiese RA, Turnquist EM. BARRIERS: The Barriers to Research Utilization Scale. Adapted from Crane, J., Pelz, D., and Horsley, JA CURN Project Research Utilisation Questionnaire. Ann Arbor, Michigan: Conduct and Utilization of Research in Nursing Project, School of Nursing. The University of Michigan. 1987.

[17] Dalheim A, Harthug S, Nilsen RM, Nortvedt MW. Factors influencing the development of evidence-based practice among nurses: a self-report survey. BMC Health Serv Res [Internet]. 2012 Jan [cited 2014 Dec 14]; 12(1):367. doi: 10.1186/1472-6963-12-367 23092366 PMC3557178

[18] Boswell C., Ashcraft A., Long J., Cannon S., DiVito‐Thomas P. and Delaney T., 2020. Self‐efficacy: Changing the tide of evidence‐based practice. *Worldviews on Evidence‐Based Nursing*, 17(2), pp.129–135. doi: 10.1111/wvn.12434 32243684

[19] Li S, Cao M, Zhu X. Evidence-based practice: Knowledge, attitudes, implementation, facilitators, and barriers among community nurses—systematic review. Medicine. 2019 Sep;98(39). doi: 10.1097/MD.0000000000017209 31574830 PMC6775415

[20] Moore S, Jayewardene D. The use of smartphones in clinical practice. Nursing Management. 2014 Jun 26;21(4). doi: 10.7748/nm.21.4.18.e1225 24967805

[21] Ammouri AA, Raddaha AA, Dsouza P, Geethakrishnan R, Noronha JA, Obeidat AA, et al. Evidence-based practice: Knowledge, attitudes, practice and perceived barriers among nurses in Oman. Sultan Qaboos University Medical Journal. 2014 Nov;14(4): e537. 25364558 PMC4205067

[22] Ma’moun AS, Abu-Moghli FA. Perceived knowledge, attitudes, and implementation of evidence-based practice among Jordanian nurses in critical care units. Dimensions of Critical Care Nursing. 2020 Sep 1;39(5):278–86. doi: 10.1097/DCC.0000000000000431 32740199

[23] Beshir MA, Woreta SA, Kebede M. Evidence-based practice among health professionals in hospitals of Northwest Ethiopia: a cross-sectional study. International journal of evidence-based healthcare. 2017 Dec 1;15(4):161–70. doi: 10.1097/XEB.0000000000000111 28509809

[24] Penz KL, Bassendowski SL. Evidence-based nursing in clinical practice: implications for nurse educators. The Journal of Continuing Education in Nursing. 2006 Nov 1;37(6):250–4. doi: 10.3928/00220124-20061101-03 17144114

